# Neuronal Representation of Object Choice in the Striatum of the Monkey

**DOI:** 10.3389/fnins.2019.01283

**Published:** 2019-11-28

**Authors:** Satoshi Nonomura, Kazuyuki Samejima

**Affiliations:** ^1^Brain Science Institute, Tamagawa University, Tokyo, Japan; ^2^Physiology and Cell Biology, Graduate School of Medical and Dental Sciences, Tokyo Medical and Dental University, Tokyo, Japan

**Keywords:** decision-making, object choice, striatum, monkey, electrophysiology, mutual information analysis

## Abstract

According to a widely held view, the decision-making process can be conceptualized as a two-step process: “object choice,” which does not include physical actions, followed by “movement choice,” in which action is executed to obtain the object. Accumulating evidence in the field of decision neuroscience suggests that the cortico-basal ganglia circuits play a crucial role in decision-making. However, the underlying mechanisms of the object and movement choices remain poorly understood, mainly because the two processes occur simultaneously in most experiments. In this study, to uncover the neuronal basis of object choice in the striatum, the main input site of the basal ganglia, we designed a behavioral task in which the processes of object and movement choice were temporally separated, and recorded the single-unit activity of phasically active neurons (PANs) (*n* = 375) in the striatum of two monkeys. We focused our study mainly on neuronal representation during the object choice period, before movement choice, using a mutual information analysis. Population striatal activities significantly represented the information of the chosen object during the object choice period, which indicated that the monkeys actually made the object choice during the task. For the activity of each individual neuron during the object choice period, we identified offered object- and chosen object-type neurons, corresponding to pre- and post-decision signals, respectively. We also found the movement-type neurons during the movement period after the object choice. Most offered object- or chosen object-type neurons were not overlapped with movement-type neurons. The presence of object choice-related signals independent of movement signal in the striatum indicated that the striatum was part of the site where object choice was made within a cortico-basal ganglia circuit.

## Introduction

We often make decisions among abstract outcomes without undertaking physical actions. For example, imagine that you are in a *kaitenzushi* restaurant (also known as conveyor-belt sushi or sushi train). You can decide on the sushi topping before reaching your hand toward the sushi on the dish carried by conveyor belt. In this case, the first step, which does not include the physical action (reaching your hand), could be regarded as the “object choice”; it is followed by the second step, the “movement choice,” in which an action is executed to obtain the object when the object is conveyed in front of you. Recently, several neuroscientists have discussed the concept and neuronal mechanism of the consecutive two-step decision processes (Samejima and Doya, 2007; Padoa-Schioppa, 2011; Cisek, 2012; Chen and Stuphorn, 2015).

The striatum, the main entry nucleus of the basal ganglia, is thought to play major roles in decision-making. Anatomically, the striatum has inputs from various cerebral cortical areas, including the prefrontal, higher-order motor, and primary motor cortex, and it returns these inputs to the cortical areas largely in parallel via the thalamus (Yeterian and Van Hoesen, 1978; Selemon and Goldman-Rakic, 1985; Alexander et al., 1986; Flaherty and Graybiel, 1993; Haber and Knutson, 2010). Clinically, patients with Parkinson’s disease, Huntington’s disease, or obsessive–compulsive disorders, all of which are considered disorders of the basal ganglia, exhibit cognitive dysfunction in action choice as well as motor behaviors (Graybiel and Rauch, 2000; Mink, 2003; Frank et al., 2004; Beste et al., 2008). Several lines of evidence from primate and rodent electrophysiological and optogenetic studies have shown that the striatum plays important roles in decision-making by predicting future goals, taking action, and monitoring performance and outcome in order to improve future behavior (Lauwereyns et al., 2002; Takikawa et al., 2002; Cromwell and Schultz, 2003; Samejima et al., 2005; Yamada et al., 2007; Lau and Glimcher, 2008; Cai et al., 2011; Tai et al., 2012; Nonomura et al., 2018).

Note that although there is considerable evidence for the neural basis of decision-making in the striatum, it remains unknown whether and how this region of the brain is involved in the consecutive two-step choice process, i.e., object and movement choice. Because most studies in primates and rodents adopted behavioral tasks in which the alternatives for choice included both motor and non-motor factors simultaneously, e.g., alternatives predicting different reward values (non-motor factor) and the direction of a moving joystick (motor factor) (Samejima et al., 2005), neuronal activity in relation to the object and movement choices could not be clearly dissociated. Several studies have reported that an object signal unrelated to movement direction to guide the choice was represented in the orbitofrontal cortex (OFC), the supplementary eye field (SEF), and the amygdala (Padoa-Schioppa and Assad, 2006; So and Stuphorn, 2010; Grabenhorst et al., 2012; Cai and Padoa-Schioppa, 2014; Chen and Stuphorn, 2015). However, few studies have investigated the neuronal representation related to object choice in the striatum.

In this study, to investigate the neuronal representation of object choice in the striatum, we designed a choice task, in which consecutive two-step choice processes were temporally decomposed, recorded single-unit activity in the striatum of macaques performing the task, and performed a mutual information analysis. This is the first study to provide an evidentiary neuronal representation of the striatum for object choice.

## Materials and Methods

### Animals and Surgery

All experiments were approved by the Animal Research Ethics Committee of Tamagawa University (animal experiment protocol H21/27-14) and were carried out in accordance with the Fundamental Guidelines for Proper Conduct of Animal Experiments and Related Activities in Academic Research Institutions (Ministry of Education, Culture, Sports, Science and Technology of Japan) and the Guidelines for Animal Experimentation in Neuroscience (Japan Neuroscience Society). All surgical procedures were performed under appropriate anesthesia, and all efforts were made to minimize suffering (see below). Our procedures for primate animal experiments were established in previous studies at Tamagawa University (Nakayama et al., 2008; Yamagata et al., 2009; Hashimoto et al., 2010; Saga et al., 2011; Arimura et al., 2013).

We used two monkeys (*Macaca fuscata*): monkey 1 (8.5 kg) and monkey 2 (8.0 kg). During the experimental sessions, each monkey sat in a chair with its head and both arms restrained and its right wrist left free to enable it to push a button with its hand; the button was installed in front of the chair at waist level. A 19-inch video monitor screen equipped with a speaker to provide sound stimulation was placed in front of the monkey. Eye positions were monitored at 240 Hz with an infrared eye-tracking system (resolution, 0.25° in visual angle; EYETRAC6000, Applied Science Laboratories). The distance between the screen and the monkey’s eyes was 340 mm. A tube was located near the monkey’s mouth to give a reward of apple juice. The amount of reward was controlled by opening and closing an electromagnetic valve via a control signal from a TEMPO system (Reflective Computing, Olympia, WA, United States), which was also used to control the behavioral task, visual stimulus presentation by the liquid crystal display, and the sound stimulus predicting the amount of reward. The order of presentation of the visual stimuli was controlled by custom MATLAB code (Math Works).

### Behavioral Task

Two tasks were designed, a free-choice task and an instruction task. While seated in the chair, the monkey performed the task by operating a push-button with its right hand according to a visual stimulus presenting the alternatives for choice. If the monkey successfully performed the task, an apple juice reward following the reinforcement sound was given. Four different amounts of reward were used (reward 1, 0.095 ml; reward 2, 0.190 ml; reward 3, 0.284 ml; and reward 4, 0.376 ml). A reinforcement sound corresponding to the amount of reward was repeated before actual delivery of the reward (one to four repetitions of a short, high tone, corresponding to one to four units of reward).

**FIGURE 1:**
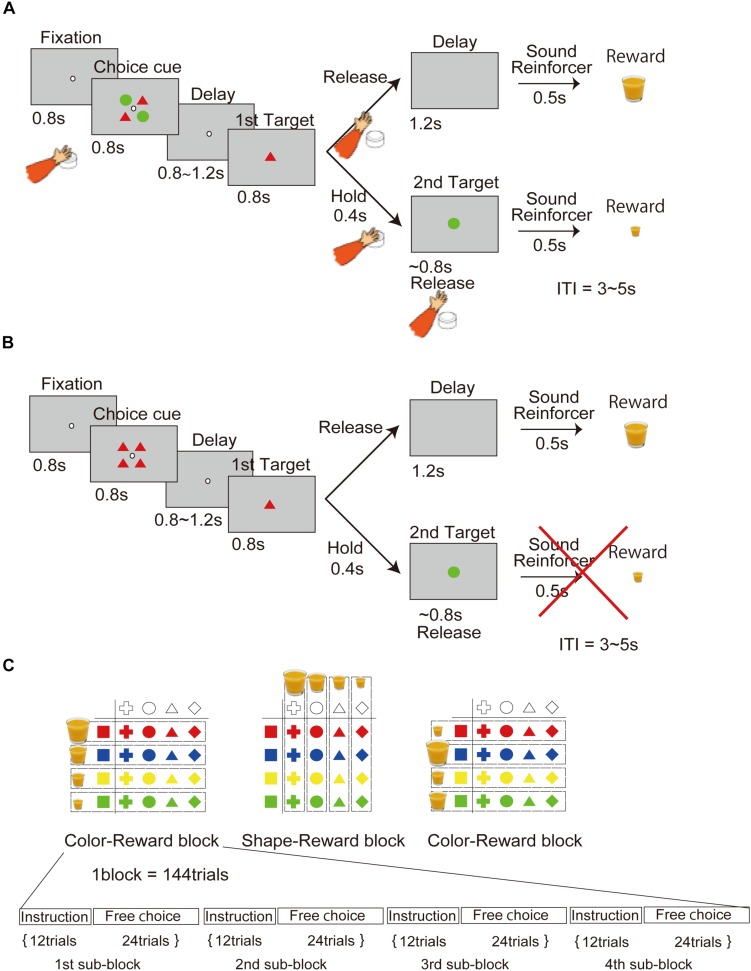
Behavioral task in which the processes of the object and movement choices are temporally dissociated. **(A)** Free-choice task. Two objects associated with different amounts of reward, without movement factors, were presented as the choice cue for release of the button at a later time. **(B)** Instruction task. An object was presented as an instruction cue instead of the choice cue in the free-choice task. **(C)** Object–reward association schedule. The association between four different amounts of reward and object of four different colors and shapes was randomly changed in each block.

In the free-choice task (Figure 1A), the monkey had to choose one of two objects presented on the screen. Pushing the button located near the monkey’s hand started the task, after which a fixation point (4.5 × 4.5 mm white square dot) appeared in the center of the screen. If the monkey maintained its gaze on the fixation point under 1 degree for 0.8 s, a choice cue was presented in a 40 × 40 mm square area (under 6.7 degrees) for 0.8 s. The choice cue consisted of two types of objects located at four corners (upper left and right, lower right and left). Each object was 20 mm in diameter. The choice cues were randomly picked from 16 objects (four colors × four shapes). After a delay period (0.8–1.2 s), two objects were individually presented again in random order as the first and second target. The monkey had to choose one of the two targets by releasing the button during presentation of the target. If the monkey released the button during the first presentation of the target (0.8 s), it received a reward of a size corresponding to the first target after a 1.2-s first-release delay period and a 0.5-s reinforcement sound. Conversely, if the monkey kept holding the button throughout the first target presentation (0.8 s), the second target was presented following a delay period of 0.4 s. If the monkey released the button during presentation of the second target (0.8 s), it received a reward of a size corresponding to the second target after a 0.5-s reinforcement sound. Trials were separated by an interval of 3–5 s. A trial was aborted if the monkey failed to maintain fixation of its gaze (over 1 degree) throughout presentation of the fixation point (0.8 s). When an aborted trial was detected, all presented objects were immediately extinguished, neither the reinforcement sound nor the reward was delivered, and the trial began again.

**FIGURE 2:**
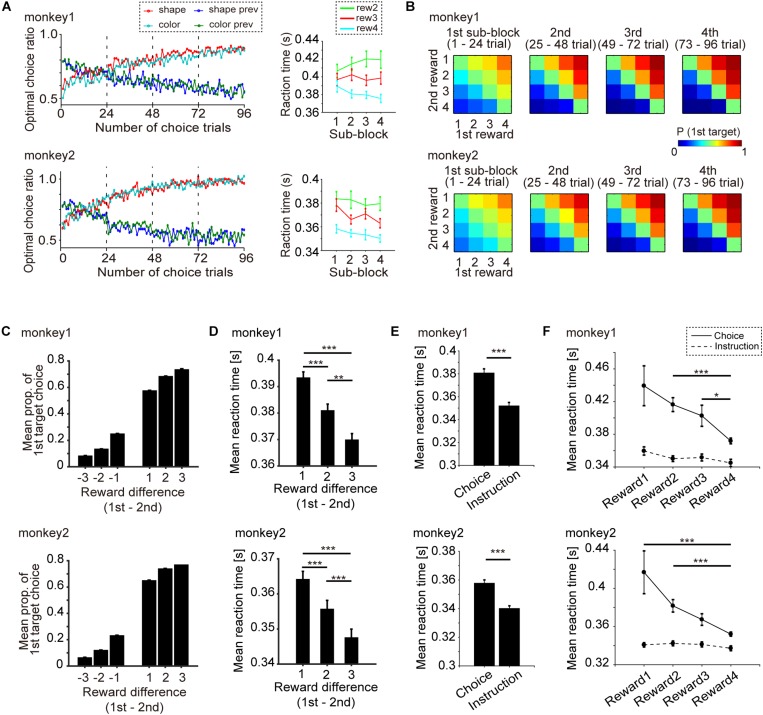
Behavioral performance (monkeys 1 and 2: 119 days, 178 blocks; monkey 2: 86 days, 129 blocks). **(A)** Left: Transition of mean optimal choice ratio in the free-choice task after the block change with monkeys 1 and 2. Red and cyan indicate the optimal choice ratio in the current shape and color block, respectively. Blue and green indicate the optimal choice ratios in the previous shape and color blocks, respectively. Right: Mean reaction time (RT) and s.e.m. of the optimal choice trial vs. progress of subblocks (1st, 2nd, 3rd, and 4th) with monkeys 1 and 2. Cyan, red and green indicate RT when the chosen amount of reward was 4, 3, and 2, respectively. **(B)** Mean probability of choosing the first target vs. the difference in the amount of reward with monkeys 1 and 2. **(C)** Mean probability of choosing the first target vs. the difference in reward amount (1st minus 2nd target) and s.e.m. for monkey 1 and monkey 2. **(D)** Mean RT and s.e.m. when the first target was chosen vs. the difference in the amount of reward (1st minus 2nd target) for monkeys 1 and 2. **(E)** Mean RT and s.e.m. of the choice task and the instruction task for monkey 1 and 2. **(F)** Mean RT and s.e.m. of the choice task and the instruction task for the four level of reward amount. Statistical test was performed by Wilcoxon rank sum test with Bonferroni correction. ^∗∗∗^*p* < 0.001, ^∗∗^*p* < 0.01, ^∗^*p* < 0.05.

In the instruction task (Figure 1B), the monkey had to choose only one instructed object presented on the screen. The task sequence was the same as in the choice task, except that in this case, the choice cue was the instruction cue (only one type object).

According to the reward schedule (Figure 1C), the task was run in a block of 144 trials consisting of the first through the fourth subblocks. Each subblock included 12 trials of the instruction task and 24 trials of the free-choice task. Four different amounts of juice reward were associated with four colors or shapes in a block of trials. The association of color with reward and shape with reward was altered block by block. The amount of reward associated with each shape or color in a block was randomly changed across every block.

### Electrophysiological Recording

After completing the behavioral training, the monkeys underwent aseptic surgery performed under pentobarbital sodium anesthesia (20–25 mg/kg, i.v.) with atropine sulfate. Antibiotics and analgesics were used to prevent postsurgical infection and pain. Polycarbonate screws were implanted in the skull, and two plastic pipes, rigidly attached with acrylic resin, were used to securely fix the head during the daily recording sessions. Part of the skull was removed over the anterior part of the striatum, and a recording chamber was implanted, tilted laterally by 35°. To confirm that the chamber was located appropriately to approach the target brain area, magnetic resonance images were recorded. Neuronal activity was recorded with glass-insulated tungsten electrodes (1.0–2 MΩ at 1 kHz; Alpha Omega Engineering) advanced by an oil-driven micromanipulator (MO-97-S; Narishige). The recording sites were determined using a grid system, which allowed us to record at intervals of 1 mm between penetrations. The electrode was introduced into the brain through a stainless steel guide tube, which was inserted into one of the grid holes and then into the brain through the dura mater. Detection of electrical signals from the electrode and online sorting were performed by a Multichannel Acquisition Processor (MAP/16, Plexon). The signal was amplified by a head-stage (HST/8o50-G20) and pre-amp with a band-pass filter (PBX2/16wb-G50, Plexon; final gain, 500; band-pass filter 0.1–8 kHz) and collected at 1 kHz. The behavioral task was controlled by a TEMPO system and MATLAB. The signals controlling the behavioral task from the TEMPO system were recorded in the MAP system with the neuronal signals. Offline sorting of action potentials was performed with an Offline Sorter (ver3, Plexon). The sorted action potentials and behavioral data were analyzed by MATLAB.

The recording site was the striatum of the left hemisphere (A: 21–30 mm and L: 18–27 mm for monkey 1; A: 22–30 mm and L: 18–28 mm for monkey 2). The dorsal border of the striatum was easily identified from changes in the background firing rate as the electrode was introduced through the cortex, white matter, and striatum. We classified striatum neurons as phasically active neurons (PANs) or tonically active neurons based on differences in spontaneous activity and spike waveform (Hikosaka et al., 1989; Aosaki et al., 1994). If we judged a PAN to be responsive to any task event by observing a phasic response during a trial, we started recording. All neurons in the database were recorded across at least two blocks of trials, including one shape-reward and one color-reward block.

### Data Analysis

To determine whether the monkey actually made an object choice during the choice cue or delay period, we adopted the latter half of the trials in the block (third and fourth subblocks) for analysis to eliminate the effect of learning about the association between reward and objects. Unless otherwise noted, we analyzed the neuronal data in the free-choice task not including the instruction task. To investigate the neuronal representation related to object choice, we performed mutual information analysis for each recorded neuron (Optican and Richmond, 1987). Mutual information for each neuron was calculated based on the difference between *a priori* information of a task condition and information of the task condition given the firing rate in the trial. The following equation was used:

$$I\,\left(S;R\right)=H\,\left(S\right)-H\,\left(S|R\right)=\sum_{s}-p\,\left(s\right)\,\log p\,\left(s\right)-{\left\langle \sum_{s}-p\,\left(s|r\right)\,\log p\,\left(s|r\right)\right\rangle }_{r}$$

where *S* is the set of task conditions {S1, S2 …}, *R* is the set of observed neuronal activities ri: the firing rate in the i–th trial, *H*(*S*) is a prior information entropy of the task condition *S*, *H*(*S*| *R*) is the information entropy of task condition *S* given neuronal activity *R* in the trial, and ⟨⟩_*r*_ is the mean information entropy across all task conditions *s* given neuronal activity *r*. Here two task conditions *S* (*S1* and *S2*) were used. The first task condition, *S1*, was six combinations of the choice cue (referred to as “offered object”), including six color or shape combinations (*S1_*color*_* = {red/blue, red/yellow, red/green, blue/yellow, blue/green, and yellow/green} and *S1_*shape*_* = {circle/triangle, circle/square, circle/cross, triangle/square, triangle/cross, and square/cross}). Under the first task condition, *S1*, *p*(*s*) was calculated using the probability of 1/6, and *p(s| r)* was calculated using the probability that trials *s* exhibit higher firing rates than the median firing rate across all trials. The second task condition, *S2*, was four colors or shapes of the chosen object (*S2_*color*_* = {red, blue, yellow, and green} and *S2_*shape*_* = {circle, triangle, square, and cross}). Under the second task condition, *S2, p*(*s*) was calculated using the probability of 1/4, and *p(s| r)* was calculated using the probability that trials *s* exhibit higher firing rates than the median firing rate across all trials.

**FIGURE 3:**
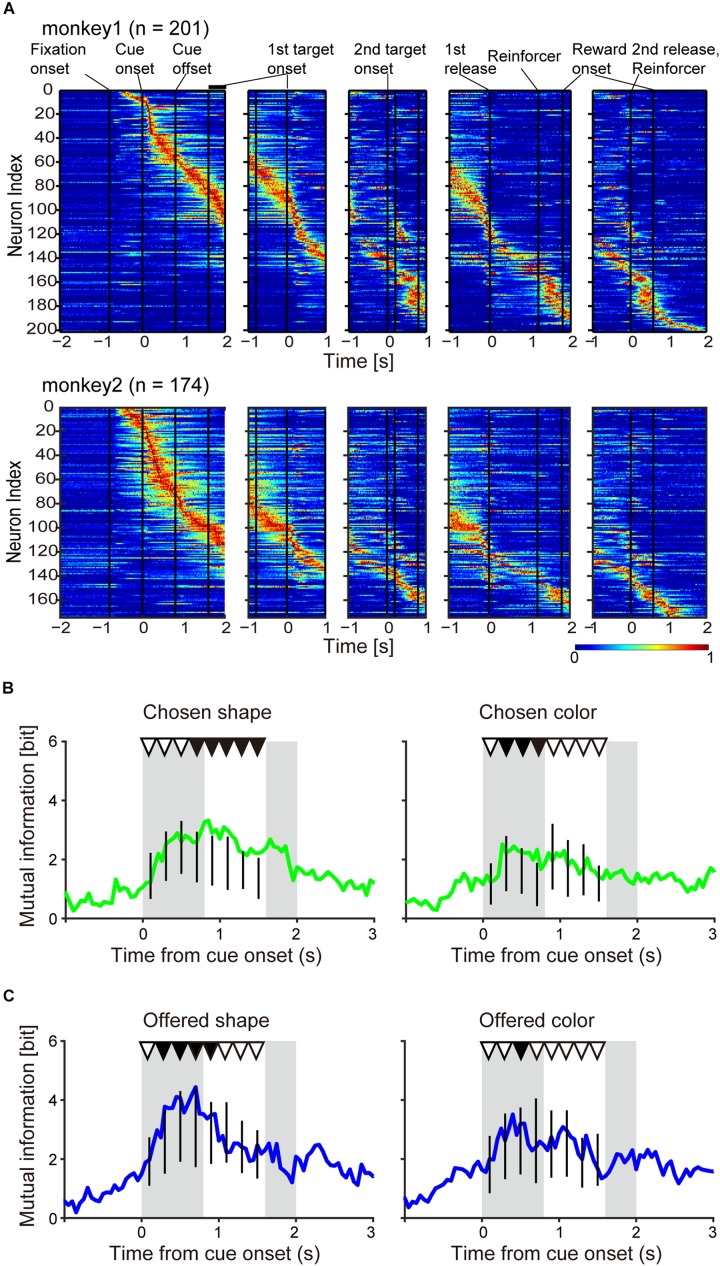
Population neuronal activity evidence for object choice during the choice cue and delay periods. **(A)** Normalized firing rate of monkeys 1 and 2. The firing rate was normalized for each neuron with the maximum firing rate in a 0.05-s window. **(B)** Time course of mutual information of chosen object of shape (left) and color (right) for all recorded neurons (*n* = 375) aligned at onset of choice cue. Vertical black lines indicate the surrogate distribution of the mutual information of chosen shape (*n*_shuffles_ = 10,000) in successive eight 0.2-s windows from onset of choice cue. First and second gray shadows indicate the cue presentation period (0.4 s) and onset of the first target following variable cue delay period, respectively. Upper black and white triangles indicate significant differences between real mutual information and surrogate information and non-significant differences between them, respectively. **(C)** Same as **(B)** but for the information of offered object.

To find evidence that the monkeys actually made an object choice before a movement choice, we calculated mutual information of the chosen object during the period from onset of choice cue to onset of the first target. Because there was the potential that mutual information of the chosen object had a spurious correlation with that of the offered object, we checked whether the mutual information of the chosen object was significantly larger than that of the information expected from the offered object. To do this, we adopted the bootstrap method for the hypothesis test and calculated the surrogate mutual information of the chosen object in which the information of the chosen object was randomized but the information of the offered object was kept. For example, to calculate the surrogate mutual information of the chosen shape for every recorded neuron, we generated trial-shuffled data in which the chosen shapes were shuffled randomly in every trial group in which the same shape combination of the offered object was presented (irrespective of their colors). We used the trial-shuffled data and calculated the surrogate mutual information of the chosen shape for every recorded neuron. Then, we calculated the summation of surrogate mutual information of the chosen shape from all recorded neurons. We performed this procedure repeatedly (10,000 shuffles) and generated the surrogate distribution of the mutual information of chosen shape. The significance level was determined at the top 5% of the surrogate distribution. If the summation of real mutual information of the chosen shape was more than the significance level in the surrogate distribution, we considered that the real information of the chosen shape was significantly larger than that of the information expected from the offered shape at the population level (*p* < 0.05). In the case of information of the chosen color, we performed the same analysis using color information instead of shape. The dynamics of the summation of real information (Figure 3B) was calculated in 0.2-s sliding windows with 0.05 steps. The bootstrap method was performed for eight consecutive 0.2-s windows starting from onset of choice cue 0–0.2-s, 0.2–0.4-s, 0.4–0.6-s, 0.6–0.8-s, 0.8–1.0-s, 1.0–1.2-s, 1.2–1.4-s, and 1.4–1.6-s, corresponding to the upper triangles in Figure 3B. As with the chosen object, the significance of mutual information of the offered object was tested (*p* < 0.05) (Figure 3C). The statistical test was the same as for the chosen object except that the surrogate mutual information of the offered object was calculated by trial-shuffled data in which the offered objects were shuffled randomly in every trial group in which the same object was chosen, e.g., when the chosen object was red, the offered objects (red/blue, red/yellow, and red/green) were randomized.

**FIGURE 4:**
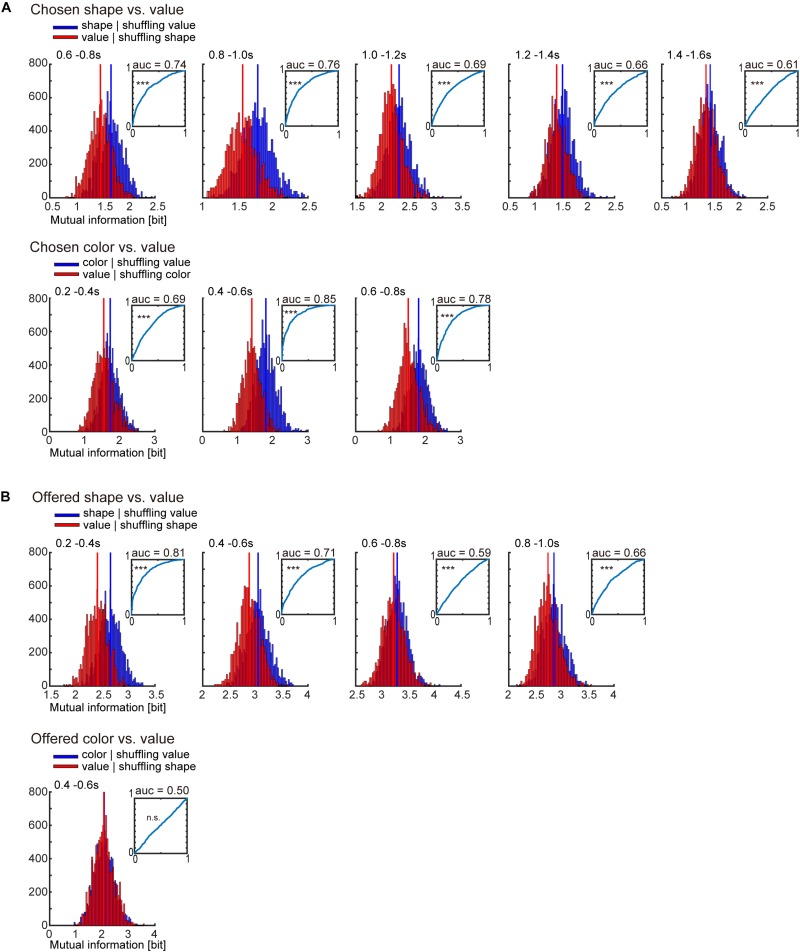
Receiver operating characteristic (ROC) analysis for classification of object-visual feature and object-reward value. **(A)** Surrogate distribution of chosen object-visual feature and object-reward value. Upper: Surrogate distribution of chosen shape (blue) and value (red) generated from shuffling of chosen value and shape, respectively (10,000 shuffles) in 0.2-s window showing significance in Figure 3B left. Inset indicates the ROC curve for classification of chosen shape. Horizontal and vertical lines indicate the false positive and true positive rates, respectively. AUC is the area under the curve. The statistical test for the AUC was performed by Mann–Whitney *U*-test (see section “Materials and Methods” for details). Lower: Surrogate distribution of chosen color (blue) and value (red) generated from shuffled chosen value and shape, respectively (10,000 shuffles) in 0.2-s window showing significance in Figure 3B right. **(B)** Same as **(A)** but for offered object (shape and color) and value. ^∗∗∗^*p* < 0.001.

To compare two surrogate distributions in Figure 4 by receiver operating characteristic (ROC) analysis for the classification of chosen (or offered) object and value, we re-calculated the two surrogate distributions and the area under the curve (AUC) ten times, and compared the AUCs with 0.5 by Mann–Whitney *U*-test.

**FIGURE 5:**
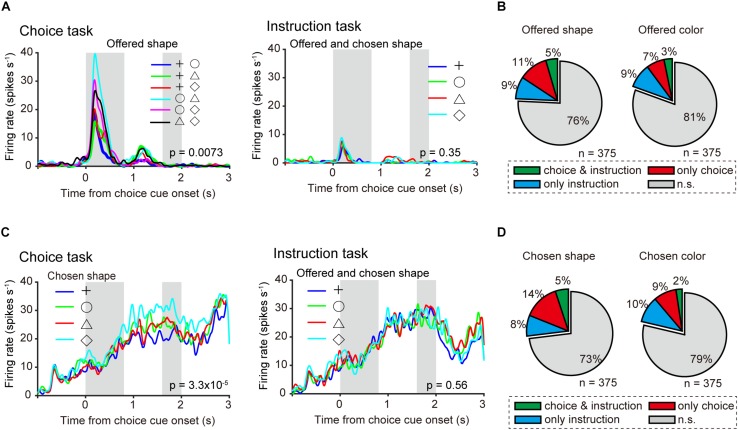
Neuronal representation in relation to object choice at the single-neuron level. **(A)** Left: Example of time course of averaged single neuronal activity representing the offered shape, defined as an offered shape-type neuron (0–0.4-s window from onset of choice cue; one-way ANOVA, *p* = 0.0073; bootstrap, *p* = 0.02). First and second gray shadows indicate cue presentation period (0.4 s) and onset of the first target following a variable cue delay period (0.8–1.2 s), respectively. Right: Firing rate, in the same neuron, sorted by offered and chosen shape in instruction task (one-way ANOVA, *p* = 0.35). **(B)** Left and right pie charts show the proportion of offered shape- and color-type neurons among all recorded neurons (*n* = 375). Green, orange, and blue indicate significant differences (*p* < 0.05) in both choice and instruction task, choice task only, and instruction task only, respectively. **(C)** Left: Example of average time course of single neuronal activity representing the chosen shape, defined as a chosen shape-type neuron (1.2–1.6-s window from onset of choice cue; one-way ANOVA, *p* = 3.3 × 10^–5^; bootstrap, *p* = 0.01). First and second gray shadows indicate cue presentation period (0.4 s) and onset of the first target following a variable cue delay period (0.8–1.2 s), respectively. Right: Firing rate, in the same neuron, sorted by offered and chosen shape in instruction task (one-way ANOVA, *p* = 0.56). **(D)** Same as **(A)** but for chosen object type neurons.

We also checked the significance of mutual information of the chosen object and the offered object at the single-neuron level (Figure 5). In this case, we compiled the surrogate distribution of mutual information (100 shuffles) in four consecutive 0.4-s windows from choice cue onset for an individual neuron, and then checked whether its real mutual information was larger than the significance level (top 5% of the distribution of surrogate mutual information, *p* < 0.05). Furthermore, we performed one-way analysis of variance (ANOVA) with factors of chosen object (color and shape) or offered object (color and shape) for four consecutive 0.4-s windows (*p* < 0.05). If both statistical tests were passed in the same window, we defined the neuron as the offered object-type (color or shape) or the chosen object type (color or shape).

**FIGURE 6:**
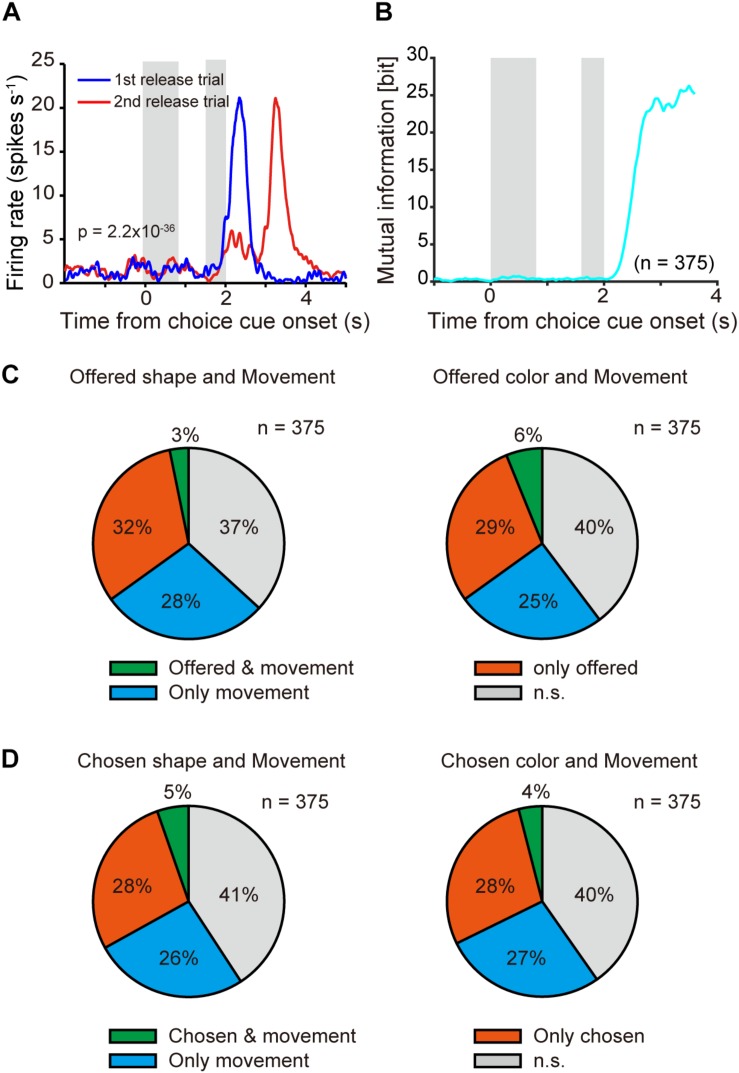
Neuronal representation in relation to movement choice. **(A)** Example of time course of averaged single neuronal activity representing movement choice (0–0.8-s window from onset of the first target; one-way ANOVA, *p* = 2.2 × 10^–36^). First and second gray shadows indicate cue presentation period (0.4 s) and onset of the first target following a variable cue delay period (0.8–1.2 s), respectively. **(B)** Time course of mutual information of movement for all recorded neurons (*n* = 375) aligned at onset of choice cue. **(C)** Overlap offered shape- (left) and color- (right) type neurons with movement-type neurons among all recorded neurons (*n* = 375). Green, orange, and blue indicate significant differences (*p* < 0.05) in both offered shape (or color) and movement, offered shape (or color) only, and movement only, respectively. **(D)** Same as **(C)** but for chosen object type neurons.

In addition to the chosen and offered object, we calculated another mutual information using the task condition of “chosen movement” (first or second release) (Figure 6B). To find movement-type neurons in Figures 6A,C,D, we performed one-way ANOVA with factor of chosen movement in the 0.8-s window from onset of the first target (*p* < 0.05).

## Results

### Behavioral Task Performance

The point of the behavioral task in this study was that object choice with a greater amount of reward during the choice cue or delay period could be made in a manner that was temporally dissociated from movement choice (Figure 1A; see section “Materials and Methods” for details). First, we confirmed that the monkeys could learn the association between a visual feature of the object (color or shape) and four levels of reward in every block of trials, and then choose the better target by releasing the button using the data recorded on neuronal activity (monkey 1, 119 days; monkey 2, 86 days) (Figure 2). The transition of the mean optimal choice ratio (the choice ratio of the target associated with the greater amount of reward) in the choice task across blocks indicated that the optimal choice ratio in both color-reward and shape-reward blocks increased after block change, whereas the optimal choice ratio corresponding to the previous block decreased in both monkeys (Figure 2A left). The reaction time (RT) from the onset of the first target to button release was faster when the monkey made an optimal choice with a larger reward than when it made an optimal choice with a smaller one, and the RTs for the larger reward became faster as the trials progressed following the block change (Figure 2A right). The monkeys could also make an optimal choice for any combination of first and second targets, predicting different amounts of reward (Figure 2B). In a quantitative analysis of the two monkeys, the choice probability of the first target and the mean RT for choosing the first target against the difference in reward amount between the first and second targets were calculated and showed consistent results with Figures 2A,B (Figures 2C,D). The choice probability of the first target against the difference exhibited a significant effect, and these choice behaviors did not differ significantly between the two monkeys (Figure 2C): two-way ANOVA, *F*(5,2) = 280.39, *p* < 0.001 for the difference in reward amount; *F*(1,6) = 0.94, *p* = 0.38 for monkey). RT was also significantly affected by the difference in reward amount (Figure 2D): one-way ANOVA, monkey 1, *F*(5,9.99), *p* < 0.001; monkey 2, *F*(1,9.16), *p* < 0.001. In both monkeys, RT was significantly slower when the difference in reward amount was 1 than when it was 2 or 3 (Wilcoxon rank sum test with Bonferroni correction: monkey 1, *p* < 0.001 for reward 1 vs. reward 2, *p* < 0.001 for reward 1 vs. reward 3, *p* = 0.0052 for reward 2 vs. reward 3; monkey 2, *p* < 0.001 for reward 1 vs. reward 2, *p* < 0.001 for reward 1 vs. reward 3, *p* < 0.001 for reward 2 vs. reward 3). These behavioral results meant that the two monkeys learned the association between a visual feature of the object (color or shape) and four different amounts of reward and chose the target associated with the greater amount of reward by releasing the button according to the block change.

Next in this task, we aimed to specifically check whether monkeys made an object choice or not (Figures 2E,F). If monkeys could choose any one of two objects during choice cue or delay period in the choice task, the subsequent process would be the same as the instruction task, in which they simply chose the decided or instructed object by release the button during target presenting period. Subsequently, we compared the RT of the choice task and the instruction task (Figures 2E,F). RT in the instruction task was found to be faster than that in the choice task (Figure 2E, Wilcoxon rank sum test: monkey1, *p* < 0.001; monkey2, *p* < 0.001). No significant effect was seen for RT in the instruction task for the four levels of reward, whereas RT in the choice task showed a significant effect (Figure 2F, one-way ANOVA, monkey1, *F*(3,1.95), *p* = 0.118 for the instruction task, *F*(3,11.4), *p* < 0.001 for the choice task; monkey2, *F*(3,0.79), *p* = 0.502 for the instruction task, *F*(3,12.1), *p* < 0.001 for the choice task). These results showing different RT between the choice and the instruction tasks indicated that the behavioral analysis was unable to support the evidence that the monkeys made object choice.

### Population Neuronal Activity Evidence for Object Choice During the Choice Cue and Delay Periods

Again, the salient feature of the behavioral task used in this study was that choice of object’s visual feature, object choice, with greater amount of reward could be made during the choice cue or delay period, temporally dissociated from movement choice. However, we were unable to confirm this claim by behavioral analysis (Figures 2E,F). It is possible that the monkeys did not always need to use a strategy to choose one of two objects during the choice cue or delay period since they could choose both object and movement at the onset of the first target by remembering the two objects without making any object choice before. In the first step of our neuronal analysis, we aimed to examine whether the monkeys actually made an object choice during the choice cue or delay period by analyzing neuronal activities of all recorded neurons (monkey 1, *n* = 201; monkey 2, *n* = 174, Figure 3A). To this end, we searched the neuronal representation of “chosen object” during the choice cue or delay period by mutual information analysis and the bootstrap method. Because there was a possibility that mutual information of the chosen object had a spurious correlation with that of the choice cue (hereafter called the “offered objects”), we tried to identify whether the mutual information of the chosen object was significantly larger than the surrogate mutual information of the chosen object in which the information of the chosen object was randomized but the information of the offered object was kept (see section “Materials and Methods” for details). We calculated the summation of mutual information of chosen shape (or color) from all recorded neurons (*n* = 375) (Figure 3B) and performed the statistical test in each eight successive 0.2-s windows from the onset of choice cue (threshold for significance: *p* < 0.05). We found significantly larger information of the chosen shape in the latter five windows and color in the second, third, and fourth windows than the surrogate information of chosen shape and color, respectively (Figure 3B and Table 1). We also checked whether there was mutual information of the offered object in distinction from surrogate mutual information of the offered object in which the information of the offered object was randomized but the information of the chosen object was kept. We found significantly larger information of the offered shape in the second to sixth windows and color in the third window than the surrogate information of offered shape and color, respectively (Figure 3C and Table 1). The significant representation of the chosen and offered object by population neuronal activities (summation of mutual information from all recorded neurons) suggested the presence of post- and pre-decision signals in the striatum along with evidence that the monkeys actually made an object choice during the choice cue or delay period prior to the movement choice.

**TABLE 1:** *P-*values for the information of chosen and offered objects related to Figure 3.

**Object**	**Type**	**Time from onset of choice cue (s)**							
		
		**0–0.2**	**0.2–0.4**	**0.4–0.6**	**0.6–0.8**	**0.8–1.0**	**1.0–1.2**	**1.2–1.4**	**1.4–1.6**
Chosen	Shape	0.486	0.058	0.090	0.006	0	0	0.002	0
	Color	0.282	0.039	0.0013	0	0.418	0.244	0.382	0.147
Offered	Shape	0.256	0	0.003	0	0.036	0.965	0.332	0.128
	Color	0.100	0.233	0.018	0.351	0.168	0.054	0.260	0.853

Many previous studies reported that the striatum represents value signals. In the present task, the association between a visual feature of the object (shape or color) and four levels of reward was changed across blocks (Figure 1C), which might enable us to discriminate the “object-visual feature” from the “reward value” associated with the object. However, there was a possibility that the change of association across blocks was not enough to discriminate them, because we recorded neuronal activity across only two or three blocks (two or three times change of the association). Then, to check whether the information of the object-visual feature and the reward value could be regarded as different or not, we compared two surrogate distributions of the summation of mutual information for all recorded neurons (*n* = 375) (Figure 4). One was the surrogate distribution of the information of chosen (or offered) reward value, in which the information of the chosen (or offered) object was randomized but the information of the chosen (or offered) reward value was kept; the other was the distribution of information of the chosen (or offered) object, in which the information of the chosen (or offered) reward value was randomized but the information of the chosen (or offered) object was kept. Comparing the two distributions by ROC analysis for the classification of chosen object and chosen reward value in 0.2-s windows showing the significance in Figure 3B, the AUC for chosen shape was 0.74, 0.76, 0.69, 0.66, and 0.61 (Figure 4A upper, *p* < 0.001 for all five windows, Mann–Whitney *U*-test for difference between the distribution of AUCs and 0.5; see section “Materials and Methods” for statistics) and AUC for chosen color was 0.69, 0.85, and 0.78 (Figure 4A lower, *p* < 0.001 for all three windows, Mann–Whitney *U*-test). For the classification of offered object and reward value, the AUC for offered shape was 0.81, 0.71, 0.59, and 0.66 (Figure 4B upper, *p* < 0.001 for all four windows, Mann–Whitney *U*-test), and for offered color it was 0.50 (Figure 4B lower, *p* = 0.115, Mann–Whitney *U-*test). These results indicated that the information of chosen shape, chosen color and offered shape were larger than that of value, whereas offered color and value were not discriminated well in the present task and data.

### Neuronal Representation in Relation to Object Choice at the Single-Neuron Level

As population neuronal activities indicating that the monkeys actually made object choices were confirmed (Figures 3B,C), we investigated the neuronal representations of offered object and chosen object during the choice cue or delay period at the single-neuron level. Perievent time histogram (PETH) of an example of averaged activity of a single neuron aligned at the onset of choice cue in a choice task (Figure 5A left) revealed differential activity according to the shape combination of the offered object in the 0–0.4-s window from the onset of choice cue (one-way ANOVA, *p* = 0.0073). The mutual information of offered shape of this neuron in the same window was significantly larger than that of surrogate information, in which the information of offered shape was randomized and the information of chosen shape was kept (bootstrap method, *p* = 0.02; see section “Materials and Methods” for statistics). Here, we defined this type of neuron as an “offered shape-type neuron.” This offered shape-type neuron did not show differential activity according to the offered shape in the instruction task (*p* = 0.35) (Figure 5A right). To confirm the distribution of offered object (shape or color)-type neurons in choice task, instruction task, or both tasks, we calculated the proportion of offered object-type neurons for the choice task and the instruction task separately. For both offered shape- and color-type neurons, the proportion for both tasks exceeded the chance level that was expected from the proportion for choice and instruction task each [*x*^2^(1) = 11.945, *p* < 0.001 for offered color, *x*^2^(1) = 6.316, *p* = 0.012 for offered shape, Chi-squared test, Figure 5B]. An example of another neuron (Figure 5C left) showed differential activity according to the shape of the chosen object in the 1.2–1.6-s window from onset of choice cue in the choice task (one-way ANOVA, *p* = 0.0068). The mutual information of chosen shape of this neuron in the same window was significantly larger than the surrogate information, in which the information of chosen shape was randomized and the information of offered shape was kept (*p* = 0.01). We defined this type of neuron as a “chosen shape-type neuron.” This chosen shape-type neuron did not show differential activity according to chosen shape in the instruction task (*p* = 0.56) (Figure 5C right). Similar to the offered object-type neurons, we calculated the proportion of chosen object-type neurons for the choice and instruction tasks separately. For chosen shape-type neurons, the proportion for both tasks exceeded the chance level that was expected from the proportion for only choice and instruction tasks, whereas the proportion for chosen color-type neurons did not [*x*^2^(1) = 10.133, *p* = 0.015 for chosen shape, *x*^2^(1) = 2.293, *p* = 0.13 for chosen color, Chi-squared test, Figure 5D]. These results indicated the presence of offered and chosen object signals at the single-neuron level during the choice cue or delay period, and these signals were represented by all three types of neurons (choice, instruction, and both tasks).

In the present task, following object choice the monkey needed to release the button during the first or second target-presenting period (Figure 1). PETH of an example of averaged activity of a single neuron aligned at onset of choice cue (Figure 6A) revealed differential activity according to first or second release in the 0.8-s window after onset of the first target (one-way ANOVA, *p* = 2.2 × 10^–36^). We defined this type of neuron as a “movement-type neuron.” Mutual information analysis using task condition of movement (1st and 2nd release) revealed that the information was not evident during the choice cue and delay period, but it was strongly represented after onset of the first target (Figure 6B). We checked the overlap between offered object-type or chosen object-type neurons with movement-type neurons (Figures 6C,D). For both offered shape- and color-type neurons, the proportion overlapping with movement-type neurons was around or below the chance level [*x*^2^(1) = 19.189, *p* < 0.001 for offered shape, *x*^2^(1) = 0.354, *p* = 0.552 for offered color, Chi-squared test, Figure 6C]. For chosen shape- or color-type neurons, similar to the offered type neurons, the proportion of overlap with movement-type neurons was around or below the chance level [*x*^2^(1) = 1.132, *p* = 0.843 for chosen shape, *x*^2^(1) = 4.772, *p* = 0.0289 for chosen color, Chi-squared test, Figure 6D]. These results indicated that the object and movement signals were represented by separate neurons.

## Discussion

To study neuronal representation in relation to object choice, which does not include physical action, in the striatum, we designed a behavioral task, in which object choice could be temporally dissociated from movement choice, and trained two monkeys in the task (Figures 1, 2). We recorded 375 striatal PANs of the two monkeys (Figure 3A). We calculated the mutual information using the task condition of the chosen object for all recorded neurons and performed statistical tests using the bootstrap method, and found that population striatal activities represented the information of the chosen object in distinction from the offered object during the choice cue and delay period, which indicated that the monkeys actually made an object choice during the task (Figure 3B and Table 1). We also found the neuronal representation of offered object in distinction from chosen object during the period (Figure 3C and Table 1). For the activity of individual neurons, we investigated the neuronal representations of the offered object and chosen object and identified offered object- and chosen object-type neurons (Figure 5). Furthermore, we also identified that the movement-type neurons discriminated between the first and second release during the first target-presenting period (Figures 6A,B). Most offered object- or chosen object-type neurons did not overlap with movement-type neurons (Figures 6C,D). These findings suggested that the presence of object choice-related signals in the striatum and their signals were represented by other neurons related to movement.

Previous studies investigated the involvement of the striatum in action choice using behavioral tasks, in which the alternatives for choice included both motor and non-motor factors simultaneously, e.g., alternatives predicting reward values and motor direction (Takikawa et al., 2002; Samejima et al., 2005; Lau and Glimcher, 2008; Tai et al., 2012). Although some studies have examined the neuronal activity of the striatum in relation to reward expectation without the motor aspect (Lauwereyns et al., 2002; Cromwell and Schultz, 2003), these behavioral tasks did not include choices of alternatives. A unique feature of this study is that object choice (choice for visual feature) could be made during the choice cue or the delay periods, which was temporally dissociated from movement choice. Furthermore, in this task, because two objects were presented in 2 × 2 form spatially in four corners, spatial information of the two objects was hashed, which means that the object choice could not be made for spatial-specific position. This is the first study to reveal the neuronal representations in the striatum in relation to object choice by designing and adopting a behavioral task, in which the period used to make an object choice is explicitly extracted.

We were unable to confirm the evidence that monkeys actually made object choice through behavioral analysis (Figures 2E,F). However, in neuronal analysis, we found that the neuronal representation of chosen object was distinct from offered object during choice cue and delay period (Figure 3B), which indicated that object choice was made. We also found the neuronal representation of offered object during the period (Figure 3C). These representations of chosen and offered object were regarded as post- and pre-decision signals without physical action, respectively. In fact, chosen shape and offered shape in Figure 3 showed a dynamically significant representation in the order of the decision process (from pre- to post-decision signals). For the color representation, we were unable to explain the temporal dynamics like shape information. Further research is required to reveal the mechanisms of different signals such as shape, color, and offered and chosen information were temporally represented and related each other.

In the present study, we found the neuronal representations of the object-visual feature (chosen shape, color, and offered shape) rather than that of the reward value (Figure 4). Although this seems like a paradoxical result in comparison with previous studies reporting value-related signals in the striatum, some previous studies (Samejima et al., 2005; Lau and Glimcher, 2008) have reported that there are lots of non-value neurons that show a differential response according to movement direction when animals make a decision, as well as value type neurons. Although there is a discrepancy between present and previous tasks regarding whether the alternatives for choice include physical action or not, the non-value neurons in previous and present studies could be interpreted as the same type of neurons that represent the option signal without value. Therefore, the results of neuronal representation for object-visual feature in this study are consistent with those of previous studies.

Anatomically, the striatum has inputs from various cerebral cortical areas, including the prefrontal, higher-order motor, and primary motor cortex, and it returns these inputs via the thalamus largely in parallel (Alexander et al., 1986). A conceptual model has been proposed, in which the prefrontal and motor loops are involved in object and movement choice, respectively (Samejima and Doya, 2007). In addition, afferent nerves from different functional cortical regions on the striatum partially converge (Yeterian and Van Hoesen, 1978; Selemon and Goldman-Rakic, 1985), and it is proposed that this convergence plays a role in integrating information across reward, cognitive, and motor functions (Haber, 2016). Several studies of primate electrophysiology have suggested that the OFC and the SEF play an important role in reward-based action choice without the motor aspect (Padoa-Schioppa and Assad, 2006; Padoa-Schioppa, 2009; So and Stuphorn, 2010; Cai and Padoa-Schioppa, 2014; Chen and Stuphorn, 2015). Non-spatial visual information about color or shape is represented in the prefrontal cortex (Divac et al., 1967; Levy et al., 1997; Sakagami and Tsutsui, 1999; Lauwereyns et al., 2001). The anterior caudate receives input mainly from the prefrontal cortex, including the dorsolateral prefrontal cortex, the OFC, the anterior cingulate cortex, and the SEF (Yeterian and Van Hoesen, 1978; Selemon and Goldman-Rakic, 1985; Alexander et al., 1986; Haber, 2016). Considering the anatomical connections from the prefrontal cortex to the anterior caudate and its neuronal representation, including the present results, object choice could be made through the prefrontal loop including the anterior caudate and the prefrontal cortex by using information about its non-spatial value and attributes of object. However, human functional magnetic resonance imaging (fMRI) studies have reported the presence of object choice signals in the ventromedial prefrontal cortex (Wunderlich et al., 2010; Hare et al., 2011), projecting mainly to the ventral striatum. The neuronal representations for the object choice in each striatal subarea need to be investigated. On the other hand, for movement choice, several studies have suggested that the premotor cortex plays an important role (Schieber, 2000; Lauwereyns et al., 2002; Nakayama et al., 2008; Thura and Cisek, 2014). The present study revealed the presence of movement-related signals (Figure 6). Considering the anatomical connections, their movement-related signals might be processed within the premotor loop. It will be necessary to classify the distribution of neuronal representation in relation to object choice and movement choice based on the striatal subregion.

Taken together, the investigation of object choice has so far concentrated on the cortex. Our results reveal the neuronal representation in relation to object choice in the striatum and show the importance of cortico-basal ganglia circuits in decision-making.

## Data Availability Statement

All data that support the findings of this study are available from the Lead Contact (satoshi.nonomura@gmail.com) upon reasonable request.

## Ethics Statement

All experiments were approved by the Animal Research Ethics Committee of Tamagawa University (animal experiment protocol H21/27-14) and were carried out in accordance with the Fundamental Guidelines for Proper Conduct of Animal Experiments and Related Activities in Academic Research Institutions [Ministry of Education, Culture, Sports, Science and Technology (MEXT) of Japan] and the Guidelines for Animal Experimentation in Neuroscience (Japan Neuroscience Society). All surgical procedures were performed under appropriate anesthesia, and all efforts were made to minimize suffering. Our procedures for primate animal experiments were established in previous studies at Tamagawa University (Nakayama et al., 2008; Yamagata et al., 2009; Hashimoto et al., 2010; Saga et al., 2011; Arimura et al., 2013).

## Author Contributions

SN and KS designed and performed the research, analyzed the data, and wrote the manuscript.

## Conflict of Interest

The authors declare that the research was conducted in the absence of any commercial or financial relationships that could be construed as a potential conflict of interest.
